# Retinoic acid receptor-related orphan receptor α regulates bystander activation of memory CD8^+^ T cells

**DOI:** 10.3389/fimmu.2025.1647746

**Published:** 2025-08-14

**Authors:** Zimeng Cai, Mina Kozai, Hironobu Mita, Hiroto Takeuchi, Satoru Mizuno, Kazuhiro Matsuo, Kensuke Takada

**Affiliations:** ^1^ Laboratory of Molecular Medicine, Faculty of Veterinary Medicine, Hokkaido University, Sapporo, Japan; ^2^ Division of Vaccinology for Clinical Development, Institute for Vaccine Research and Development, Hokkaido University (HU-IVReD), Sapporo, Japan

**Keywords:** T cells, immunological memory, bystander activation, inflammation, nuclear receptor

## Abstract

**Background:**

Memory CD8^+^ T cells sense inflammation and rapidly produce interferon-γ (IFN-γ) independent of cognate antigens. This innate-like property, called bystander activation, is involved in early host defense before the antigen-specific memory response. However, the molecular mechanisms underlying this activation remain unknown. Retinoic acid receptor-related orphan receptor α (RORα) belongs to the nuclear receptor family and regulates gene transcription in ligand-dependent manner. Although RORα is highly expressed in memory CD8^+^ T cells, its functional relevance has not been investigated.

**Methods:**

Primary and secondary memory T cells that are sufficient or deficient of RORα were induced by adoptive transfer of naïve OT-I T cells to recipient mice and subsequent infection with *Listeria monocytogenes* expressing ovalbumin (LM-OVA). RORα expression in memory T cells was examined by quantitative PCR. The target genes of RORα in memory T cells were explored by RNA-sequencing and verified by RORα overexpression in postactivated T cells. The impact of RORα-deficiency on bystander activation was assessed by stimulating memory T cells with inflammatory cytokines *in vitro* or injecting lipopolysaccharide (LPS) into mice bearing memory T cells.

**Results:**

RORα expression was remarkably elevated in secondary memory CD8^+^ T cells along with the enrichment of effector-like memory T cells. RORα primarily acted as a transcription factor in regulating the gene expression of the TL1A receptor. RORα deficiency abrogated the IFN-γ production by memory CD8^+^ T cells in response to IL-12 + TL1A *in vitro* and diminished the bystander response to LPS-induced inflammation *in vivo*.

**Conclusion:**

This study revealed a regulatory mechanism of bystander activation. The findings also improve our understanding of how memory T cells increase their immediate protective capacity through repeated infections and vaccinations.

## 1 Introduction

The αβ T cells contribute to host defense through an antigen-specific immune response. The resulting memory T cells rapidly proliferate and exert effector functions when they re-encounter the cognate antigen, thereby providing enhanced protection against reinfection (1–3). Memory CD8^+^ T cells have also been shown to respond to inflammatory cytokines, such as interleukin (IL)-12, IL-15, and IL-18, and mount effector responses, including interferon (IFN)-γ production, even in the absence of cognate antigens (4–11). This innate-like property, called bystander activation, is beneficial for early defense against pathogens (even antigenically irrelevant ones), orchestrating the subsequent immune response in the host (4, 8–13). However, bystander-activated CD8^+^ T cells can also mediate immunopathology under certain circumstances (14–16). The capacity to undergo bystander activation varies among memory CD8^+^ T cell subsets and increases with repeated antigen encounters (17, 18). Furthermore, the ability to sense inflammation for bystander activation can be associated with the variable expression of cytokine receptors (10, 17, 19). However, the molecular mechanisms underlying bystander activation of memory CD8^+^ T cells remain unclear.

Retinoic acid receptor-related orphan receptor α (RORα), belonging to the nuclear receptor family, regulates gene transcription in ligand-dependent manner. While RORα is known to regulate circadian rhythms (20, 21), its role in the immune system is underexplored. RORα acts synergistically with RORγt, another member of the ROR subfamily, to induce the differentiation of IL-17-producing CD4^+^ T cells (Th17) by trans-activating the IL-17 gene promoter (22). RORα is also essential for the development of type 2 innate lymphoid cells (23). Although recent reports have suggested that RORα might be involved in cholesterol metabolism, its role in CD8^+^ T cells is poorly understood (24, 25). RORα expression in CD8^+^ T cells is drastically upregulated after antigen recognition (25), peaking during contraction and remaining elevated in the memory phase. The lack of functional RORα caused by a natural mutation slightly alters the balance of effector CD8^+^ T cell subpopulations (25). However, the impact of RORα deficiency on memory CD8^+^ T cells has not been investigated.

In this study, we aimed to elucidate the relevance of high RORα expression in memory CD8^+^ T cells. RORα expression was dramatically elevated in the secondary memory CD8^+^ T cells formed after repetitive antigen encounter. RORα deficiency abolished the bystander IFN-γ production that was synergistically induced by IL-12 and tumor necrosis factor (TNF)-like ligand 1A (TL1A). Our results demonstrated that RORα directly activates the gene expression of death receptor 3 (DR3), a TL1A receptor, and regulates bystander activation of memory CD8^+^ T cells by determining their sensitivity to inflammation.

## 2 Materials and methods

### 2.1 Mice

OT-I–transgenic (26), B6.SJL-*Ptprc^a^* (B6-Ly5.1) (27), and *Rora^sg^* mice (28) have been described. C57BL/6 mice were purchased from Japan SLC. The mice were bred and maintained under specific pathogen-free conditions in our animal facility. All animal experiments were performed with approval from the Institutional Animal Care and Use Committee of Hokkaido University (approval numbers 16-0131, 20-0172, and 25-0042).

### 2.2 Flow cytometry and cell sorting

Cells were incubated with an anti-CD16/CD32 antibody (Biolegend) to block Fc receptors, and then stained with fluorochrome-labeled monoclonal antibodies (Biolegend or eBioscience) for 30 min on ice. Dead cells were stained by adding propidium iodide (Dojindo) before analysis. Data were acquired on FACSVerse (BD Biosciences) or CytoFLEX (Beckman Coulter) and analyzed using FlowJo software (TreeStar). Intracellular staining of IFN-γ and granzyme B was performed using the fixation and permeabilization buffer set (Thermo Fisher Scientific) according to the manufacturer’s instructions. For cell isolation, the cells were first incubated with fluorescein isothiocyanate-conjugated antibodies, and then the antibody-bound cells were depleted using the magnetic beads and columns (Miltenyi Biotec). The cells were subsequently sorted using FACSAria II (BD Biosciences) or CytoFLEX SRT (Beckman Coulter).

### 2.3 Bone marrow chimeras

Bone marrow cells were depleted of T cells using anti-CD90.2-conjugated magnetic beads and columns (Miltenyi Biotec). The CD45-congenic recipient mice were injected intraperitoneally with 600 μg busulfan (Otsuka Pharmaceutical) (29). On the next day, the mice were injected intravenously with 1 × 10^7^ bone marrow cells to create chimeras that were used in experiments 10–14 weeks after the bone marrow transplantation. The cells derived from the donor bone marrow were identified based on the expression of CD45.1 and CD45.2 congenic markers for isolation.

### 2.4 Adoptive T-cell transfer and *Listeria monocytogenes* infection

CD44^lo^ naïve OT-I T cells isolated from the spleens of donor mice were adoptively transferred into CD45-congenic recipient mice (2 × 10^4^ cells per recipient). The next day, the recipient mice were intraperitoneally injected with an *ActA*–deficient attenuated strain of *Listeria monocytogenes* expressing ovalbumin (LM-OVA) (5 × 10^6^ colony-forming units per mouse) (30). At 2 months post-infection, primary memory OT-I T cells were sorted from the spleens and transferred into new recipients (2 × 10^4^ cells per recipient). The secondary recipients were then infected with attenuated LM-OVA as described above to generate the secondary memory T cells.

### 2.5 RNA-sequencing

Total RNA was extracted using the RNeasy Plus Micro kit (Qiagen), following the manufacturer’s instructions. RNA sequencing analysis was performed using a contracted service by DNAFORM. The quality of the total RNA was assessed using a Bioanalyzer (Agilent) to ensure the RIN (RNA integrity number) was over 7.0. After poly (A) + RNA enrichment using the NEBNext Poly(A) mRNA Magnetic Isolation Module (New England BioLabs), double-stranded cDNA libraries (RNA-seq libraries) were prepared using the SMARTer Stranded Total RNA Sample Prep kit (Takara Bio) according to the manufacturer’s instructions. RNA-seq libraries were sequenced using paired-end reads (50 and 25 nts of reads 1 and 2, respectively) on a NextSeq 500 instrument (Illumina). The obtained raw reads were trimmed and quality-filtered using the Trim Galore (version 0.4.4), Trimmomatic (version 0.36), and cutadapt (version 1.16) software. The trimmed reads were then mapped to the murine GRCm38 genome using STAR (version 2.7.2b). Unique-mapped reads on annotated genes were counted using featureCounts (version 1.6.1). FPKM values were calculated from the mapped reads by normalizing to total counts. A pseudo-FPKM count of 0.1 was assigned to each gene to calculate the fold changes and z-scores. The genes exhibiting twofold or higher expression differences between the averages of each group were extracted and classified into clusters using the MeV software (version 4.8.1). Gene ontology analysis was performed using the DAVID online tool (version 6.8).

### 2.6 Quantitative polymerase chain reaction

The RNA was reverse-transcribed using the PrimeScript RT master mix (Takara Bio). Real-time PCR analysis was performed using TB Green Premix Ex Taq II (Takara Bio) on a LightCycler 96 System (Roche Diagnostics). The primer sequences are listed in **Supplementary Table 1**. The expression levels of the target genes were normalized to those of glyceraldehyde-3-phosphate dehydrogenase and compared with the expression levels of the control samples.

### 2.7 RORα overexpression

The genes encoding RORα (NM_001289916.1) were cloned into the retroviral vector pMXs-IRES-GFP (Cell Biolabs). For ChIP, the sequence encoding the Active Motif (AM)-tag was additionally cloned downstream of the RORα sequence. The AM-tag sequence, preceded by a start codon without RORα sequence, was cloned into pMXs-IRES-GFP to generate the tag-only control vector. The construct was transfected into the Platinum-E cell line (Cell Biolabs) using FuGene HD (Promega) to obtain the supernatant containing the retrovirus. The splenocytes from the OT-I–transgenic mice were cultured in the presence of 10 nM OVA peptide (residues 257–264, SIINFEKL, OVAp)(Anaspec) and 20 ng/mL IL-2 (Miltenyi Biotech) for 2 days. The viable cells were enriched using Lympholyte-M (Cedarlane) and incubated in Platinum-E supernatant containing retrovirus supplemented with 4 μg/mL polybrene (Nacalai Tesque). The samples were centrifuged at 2000 g and 32°C for 2 h. The cells were collected on the next day.

### 2.8 Chromatin immunoprecipitation sequencing

The sorted GFP^+^CD8^+^ cells (8 × 10^6^ cells/condition) were fixed with 1% formaldehyde for 10 min at room temperature. Then, 1.5 M glycine was added to the cells, followed by a 5-min incubation to stop the reaction. Subsequent sample treatment and sequencing analysis were included in the contract service of DNAFORM as follows. Chromatin digestion and immunoprecipitation were performed using the SimpleChIP Plus Enzymatic Chromatin IP Kit (Cell Signaling) with anti-AM-tag antibody (Active Motif). Sequencing libraries were prepared from the ChIP and Input DNAs using the SMARTer ThruPLEX Tag-seq Kit (Clontech) according to the manufacturer’s protocol. Single-read sequencing (150 bp) was performed on the DNBSEQ-G400RS sequencer (MGI Tech). Mapping and peak calls were conducted using the ENCODE ChIP-seq pipeline (https://github.com/ENCODE-DCC/chip-seq-pipeline). Reads were mapped to the mm10 reference sequence using Bowtie2 (ver. 2.3.4.3). The duplicate reads were removed with Picard (2.20.7) and samtools (1.9). Peak calling was performed using SPP (1.15.5) with default parameters. After removing the blacklisted regions, the consistency of the peaks was tested at the irreproducible discovery rate (IDR) using IDR (2.0.4.2).

### 2.9 *In vitro* stimulation of memory T cells

To analyze the response of memory T cells to cognate antigens, whole splenocytes containing donor-derived memory OT-I T cells were incubated with 0.1 nM OVAp for 3 h at 37°C in the presence of a protein transport inhibitor cocktail (Thermo Fisher Scientific) and then processed for flow cytometric analysis to assess IFN-γ production. The bystander response of memory T cells was examined by culturing the whole splenocytes for 48 h at 37°C in the presence or absence of 5 ng/mL IL-12, 50 ng/mL IL-18, and 50 ng/mL TL1A (all from Biolegend). The protein transport inhibitor cocktail was added 4 h before the end of the culture period for the intracellular staining of IFN-γ and granzyme B. The memory OT-I T cells were identified based on the expression of CD45.1 and CD45.2 in the CD8^+^ gate.

### 2.10 Bystander activation by lipopolysaccharides *in vivo*

*In vivo* induction of the bystander response was performed as previously described with minor modifications. LPS from the *Escherichia coli* O111:B4 (Sigma-Aldrich) was dissolved in phosphate-buffered saline and administered intravenously to mice at a dose of 100 μg/mouse. The spleens were harvested 4 h after the LPS injection and processed on ice for cell surface staining. The intracellular staining of IFN-γ was performed as described earlier.

### 2.11 Statistical analysis

Statistical significance was evaluated using the two-tailed unpaired or paired Student’s *t*-test. Analysis was performed with the GraphPad Prism 9.0 and Microsoft Excel software. In all figures, the *p*-values less than 0.05, 0.01, and 0.001 are shown as *, **, and ***, respectively.

## 3 Results

### 3.1 RORα expression correlates with the effector-like phenotype in memory CD8^+^ T cells

**Figure 1:**
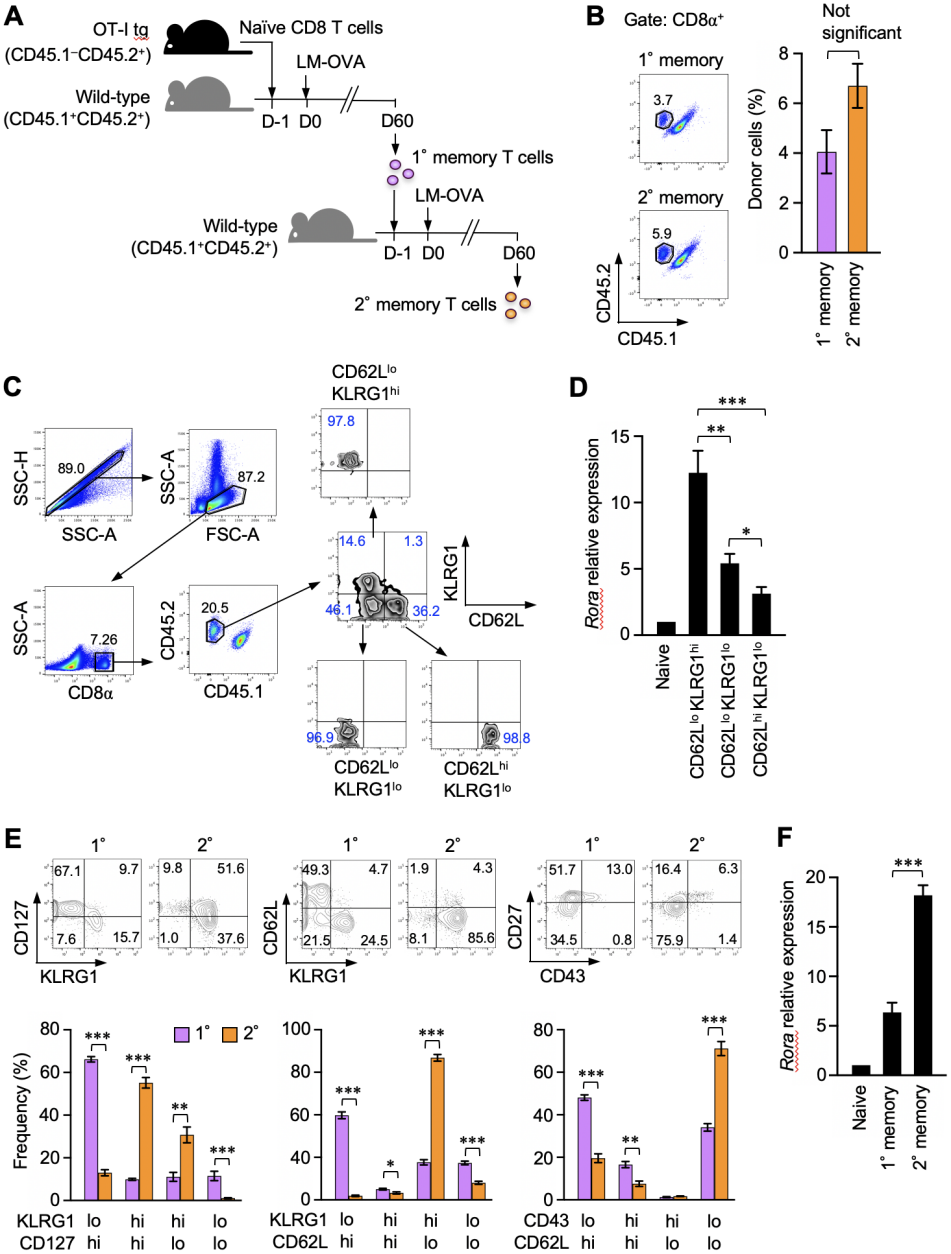
Retinoic acid receptor-related orphan receptor α (RORα) expression correlates with the effector-like phenotype in memory CD8^+^ T cells. **(A)** A representation of the experiment procedure used to obtain the primary and secondary memory CD8^+^ T cells. CD44^lo^ naïve CD8^+^ T cells (CD45.1^–^CD45.2^+^) isolated by sorting from the spleens of donor OT-I–transgenic mice were adoptively transferred into naïve CD45.1^+^CD45.2^+^ recipient mice. On the next day, the recipients were infected with *ActA*-deficient attenuated *Listeria monocytogenes*-expressing ovalbumin (LM-OVA). Sixty days later, primary memory OT-I T cells were isolated by sorting from the recipient spleen and further transferred to new recipients, who were similarly infected with LM-OVA to induce secondary memory OT-I T cells. **(B)** Primary and secondary memory OT-I T cells in the recipient spleens were identified based on the expression of CD45.1 and CD45.2 from CD8^+^ cells (n = 6). Graph shows the frequency of memory OT-I T cells in the CD8^+^ gate. **(C)** Sequential gating for the isolation of three major primary memory T cell subpopulations from the spleens (day 30 post-infection) based on the expression of CD62L and KLRG1. Propidium iodide staining were avoided specifically for sorting. **(D)** Quantitative polymerase chain reaction (qPCR) analysis of *Rora* expression in the indicated cell subsets (n = 5). The expression levels were normalized to those of glyceraldehyde-3-phosphate dehydrogenase and shown as relative to the expression levels measured in the CD44^lo^ naïve OT-I T cells defined as 1. **(E)** Phenotypic comparison of the primary and secondary memory CD8^+^ T cells based on the indicated surface markers (day 60 post-primary and secondary infection). **(F)** qPCR analysis of *Rora* expression in the primary and secondary memory T cells (n = 6). Expression data were normalized and as described in panel **(D)**, Representative **(C)** and cumulative results of three independent experiments **(B, E, F)**. Error bars, mean ± SEM. *P < 0.05, ** *P* < 0.01 and *** *P* < 0.001 (unpaired Student’s *t*-test).

Memory CD8^+^ T cells are functionally and phenotypically heterogeneous. RORα expression in different memory CD8^+^ T cell subsets was initially assessed in this study. Naïve OT-I T-cell receptor–transgenic CD8^+^ T cells, which react with the OVAp, were adoptively transferred to recipient mice. The mice were infected with LM-OVA to activate the donor OT-I T cells and induce their differentiation into primary memory T cells (**Figure 1A**). The resulting memory CD8^+^ T cells were identified based on CD45 congenic markers and divided into three subsets based on the expression of CD62L and killer cell lectin-like receptor G1 (KLRG1) (**Figures 1B, C**). The *Rora* mRNA levels were significantly higher in the CD62L^lo^KLRG1^hi^ effector-like memory T cells, while they were the lowest in the CD62L^hi^KLRG1^lo^ central memory T cells among all the subsets (**Figure 1D**). A previous study reported that secondary memory CD8^+^ T cells predominantly comprise the effector-like cells, unlike the primary memory T cells, which consist of multiple subpopulations (31). In that report, the secondary memory T cells were generated via repeated infections in the same host (31). Herein, we induced the formation of these cells by transferring the primary memory T cells into naïve mice that were subsequently infected with LM-OVA (**Figure 1A**). Nevertheless, the secondary memory CD8^+^ T cells obtained in this study were remarkably enriched with the CD127^int^CD62L^lo^KLRG1^hi^CD43^lo^CD27^lo^ effector-like subpopulation (**Figure 1E**) and expressed significantly higher levels of *Rora* mRNA than the primary memory T cells (**Figure 1F**). These results show the relevance of RORα expression to the effector-like phenotype in memory CD8^+^ T cells.

### 3.2 RORα-deficient memory CD8^+^ T cells are normal in number and antigen-specific responses

**Figure 2:**
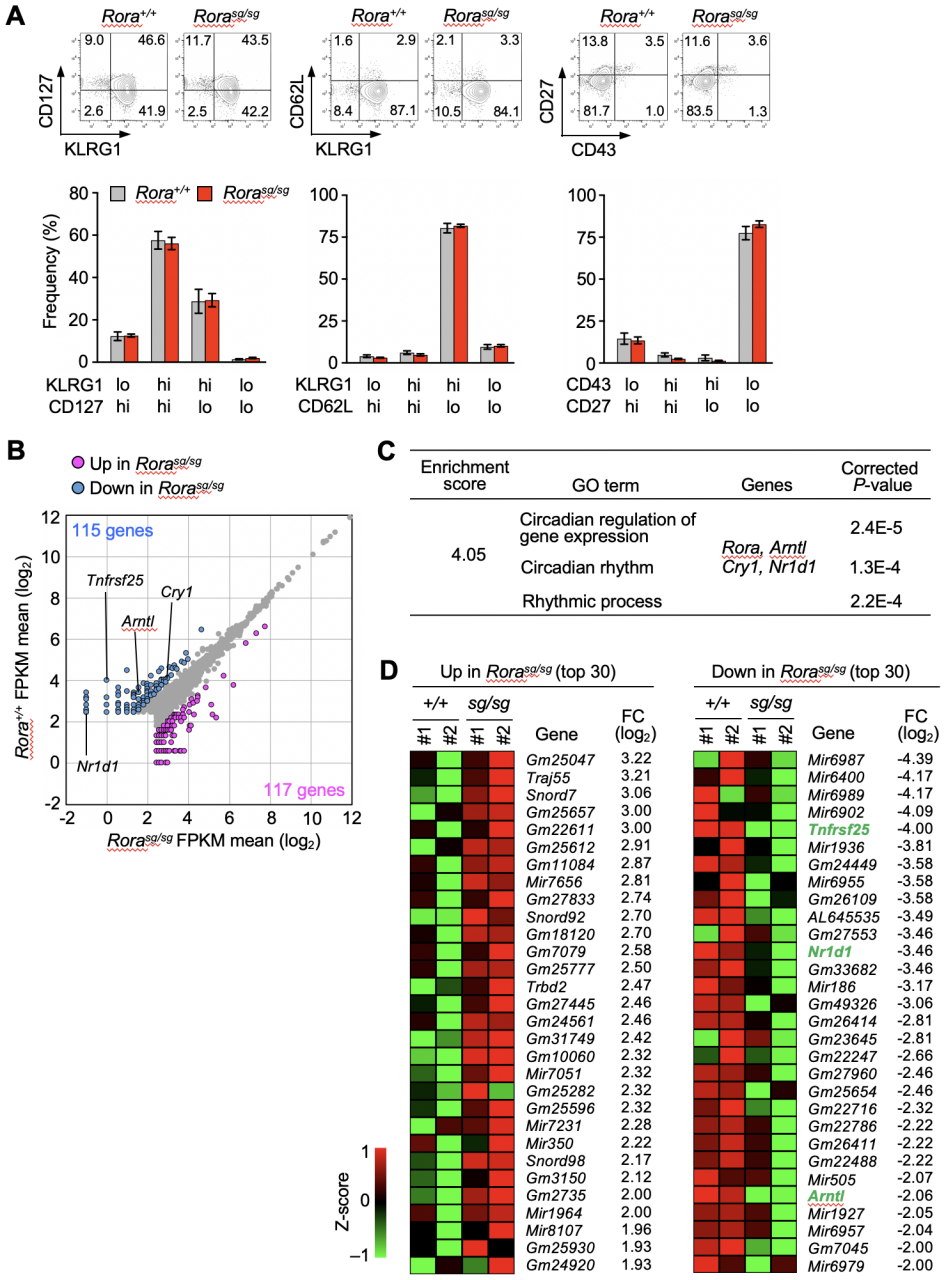
RNA-sequencing analysis of *Rora*-deficient secondary memory CD8^+^ T cells. **(A)** Bone marrow chimeras were generated by transferring the bone marrow cells from *Rora^sg/sg^* and *Rora^+/+^* OT-I–transgenic littermates into the CD45-congenic wild-type recipient mice. *Rora^sg/sg^* and *Rora^+/+^* naïve OT-I T cells were isolated from the spleens of the respective chimeras, and secondary memory T cells were induced as shown in **Figure 1A**. The phenotypes of *Rora^sg/sg^* and *Rora^+/+^* secondary memory OT-I T cells in the recipient spleens (*Rora^+/+^*, n = 8; *Rora^sg/sg^*, n = 7) were analyzed based on the indicated surface markers. Bar graphs show the frequency of each cell fraction within the secondary memory OT-I T cells. **(B–D)** RNA-sequencing analysis of *Rora^+/+^* and *Rora^sg/sg^* secondary memory T cells (n = 2/group). **(B)** The scatter plot was drawn based on the mean FPKM values from two mice per group, with the cut-off value was set to 5. The genes exhibiting twofold or higher expression in *Rora^+/+^* cells (blue dots) or in *Rora^sg/sg^* cells (magenta dots) are highlighted. **(C)** Gene ontology (GO) analysis was performed using the differentially expressed genes between the *Rora^+/+^* and *Rora^sg/sg^* groups. A list of GO terms and specifically enriched genes is shown. **(D)** The top 30 genes upregulated (left) or downregulated (right) in *Rora^sg/sg^* compared with *Rora^+/+^* cells were extracted based on the fold change (FC). Heatmaps indicate the expression of each gene in two mice per group. Compiled **(A–D)** data from two independent experiments.

The effect of RORα deficiency on the primary and secondary memory CD8^+^ T cells was examined. The OT-I transgenic mice were crossed with mice bearing a *stagger (sg)* mutation, which results in a non-functional RORα protein lacking the C-terminal ligand-binding domain. Homozygous mutants die at young age because of neurological defects (32) before the peripheral T cell pool is fully established. Therefore, bone marrow chimeras were generated by transferring the bone marrow cells from *Rora^sg/sg^* and *Rora^+/+^* OT-I–transgenic littermates into the wild-type mice. The naïve OT-I T cells were isolated from these bone marrow chimeras after the mature T cell pool was established. Then, the primary and secondary memory T cells were induced following the procedure shown in **Figure 1A**. No significant difference was observed between the *Rora^sg/sg^* and control groups in the absolute numbers of primary and secondary memory T cells (**Supplementary Figure 1A**). The overall phenotype of primary (**Supplementary Figure 1B**) and secondary (**Figure 2A**) memory T cells was unaffected by RORα deficiency. Additionally, the recall responses to antigen stimulation or infection, including the proliferative response and the production of effector molecules, were also similar between RORα-deficient and control memory T cells (**Supplementary Figures 2A–E**).

### 3.3 RORα regulates the expression of death receptor 3 in CD8^+^ T cells

The physiological functions of RORα mainly include activating the transcription of target genes (20, 21). Based on the above results that the numbers, phenotypes, and antigen-specific functions of memory T cells were unaffected by RORα-deficiency, we performed RNA-sequencing to comprehensively explore the genes in secondary memory CD8^+^ T cells. Based on the average fragments per kilobase of transcript per million mapped fragments (FPKM) values in each group, 115 and 117 genes were found to be downregulated and upregulated, respectively, in *Rora^sg/sg^* secondary memory T cells (**Figure 2B**). Gene ontology analysis based on the biological processes revealed a cluster among the downregulated genes that was enriched with clock genes, such as Aryl hydrocarbon receptor nuclear translocator like 1 (*Arntl)*, nuclear receptor subfamily 1 group D member 1 (*Nr1d1)* and cryptochrome circadian regulator 1 (*Cry1)* (**Figure 2C**). No significant enrichment of genes associated with specific biological processes was seen among the upregulated genes. Notably, the gene encoding tumor necrosis factor receptor superfamily 25 (TNFRSF25, also known as DR3) was markedly downregulated in the *Rora^sg/sg^* secondary memory T cells compared to *Arntl* and *Nr1d1* which are known as direct RORα targets in terms of fold change (33) (**Figure 2D**). *Tnfrsf25* showed the biggest fold change among downregulated genes in the RORα-deficient KLRG1^+^ effector T cells isolated at days 7 (**Supplementary Figure 3**) and 10 (**Supplementary Figure 4**) after primary infection.

**Figure 3:**
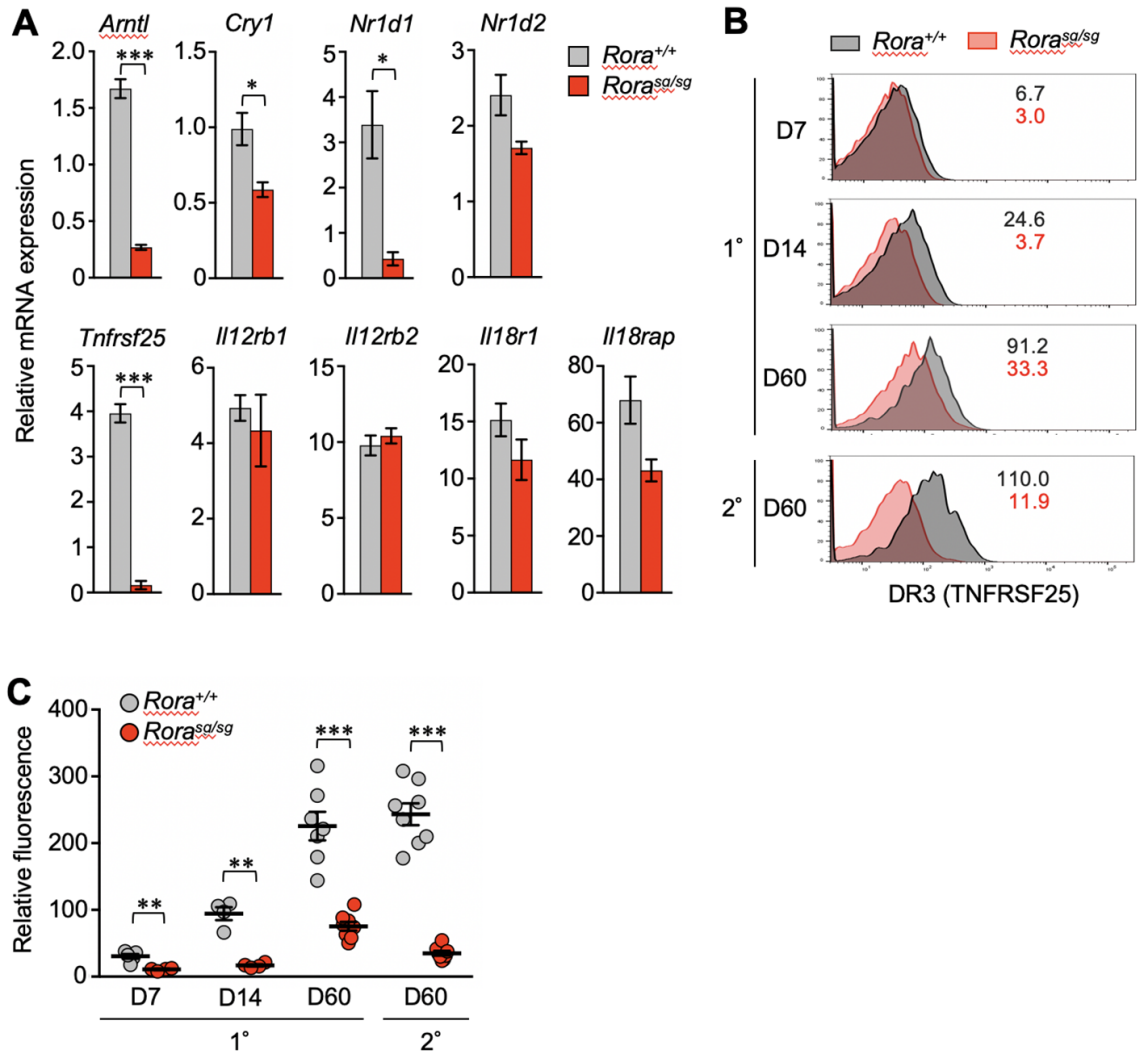
The effect of RORα deficiency on the expression of death receptor 3 (DR3) in CD8^+^ T cells. **(A)** Quantitative PCR (qPCR) analysis of relevant genes in *Rora^+/+^* and *Rora^sg/sg^* secondary memory T cells (n = 3/group). The expression levels of target genes are shown relative to those in the naïve OT-I T cells, which is defined as 1. **(B, C)** Primary and secondary responses of *Rora^+/+^* and *Rora^sg/sg^* OT-I T cells that were induced as shown in **Figure 1A**. The cell surface expression of DR3 was analyzed in donor OT-I T cells by flow cytometry on days 7, 14 and 60 post-primary infection and day 60 post-secondary infection. **(B)** The numbers in the histograms indicate the mean fluorescence intensity of DR3 in each group. **(C)** DR3 expression levels at different time points were compared according to the relative fluorescence normalized to the mean fluorescence intensity in the recipient-derived naïve (CD44^lo^) CD8^+^ T cells, which was defined as 100 (*Rora^+/+^*, n = 4–8; *Rora^sg/sg^*, n = 4–8 at each time point). Data are representative of two independent experiments **(B)**. Cumulative results from two **(C)** and three **(A)** independent experiments. Error bars, mean ± SEM. *P < 0.05, **P < 0.01 and ***P < 0.001 (unpaired Student’s *t*-test).

qPCR analysis verified that the *Tnfrsf25* mRNA levels were reduced in the *Rora^sg/sg^* secondary memory T cells (**Figure 3A**). Diminished expression of clock genes such as *Arntl*, *Cry1, and Nr1d1* was also observed, consistent with the RNA-sequencing results (**Figures 2B–D**). TL1A (also known as TNFSF15), a specific ligand of DR3, has been shown to induce bystander cytokine production from effector CD4^+^ T cells synergistically with IL-12 and IL-18 (34, 35). Therefore, we analyzed the expression of genes encoding the IL-12 and IL-18 receptors by qPCR but found no significant effect of RORα deficiency on these genes (**Figure 3A**).

In the control *Rora^+/+^* T cells, the cell surface expression of DR3 increased over time after the primary infection from the expansion to memory phases. The expression was highly maintained in secondary memory T cells (**Figures 3B, C**). However, DR3 expression was almost completely abolished due to RORα deficiency in effector T cells (1–2 weeks after primary infection) and secondary memory T cells (**Figures 3B, C**). Notably, *Rora^sg/sg^* primary memory T cells retained a low but stable level of DR3 (**Figures 3B, C**).

**Figure 4:**
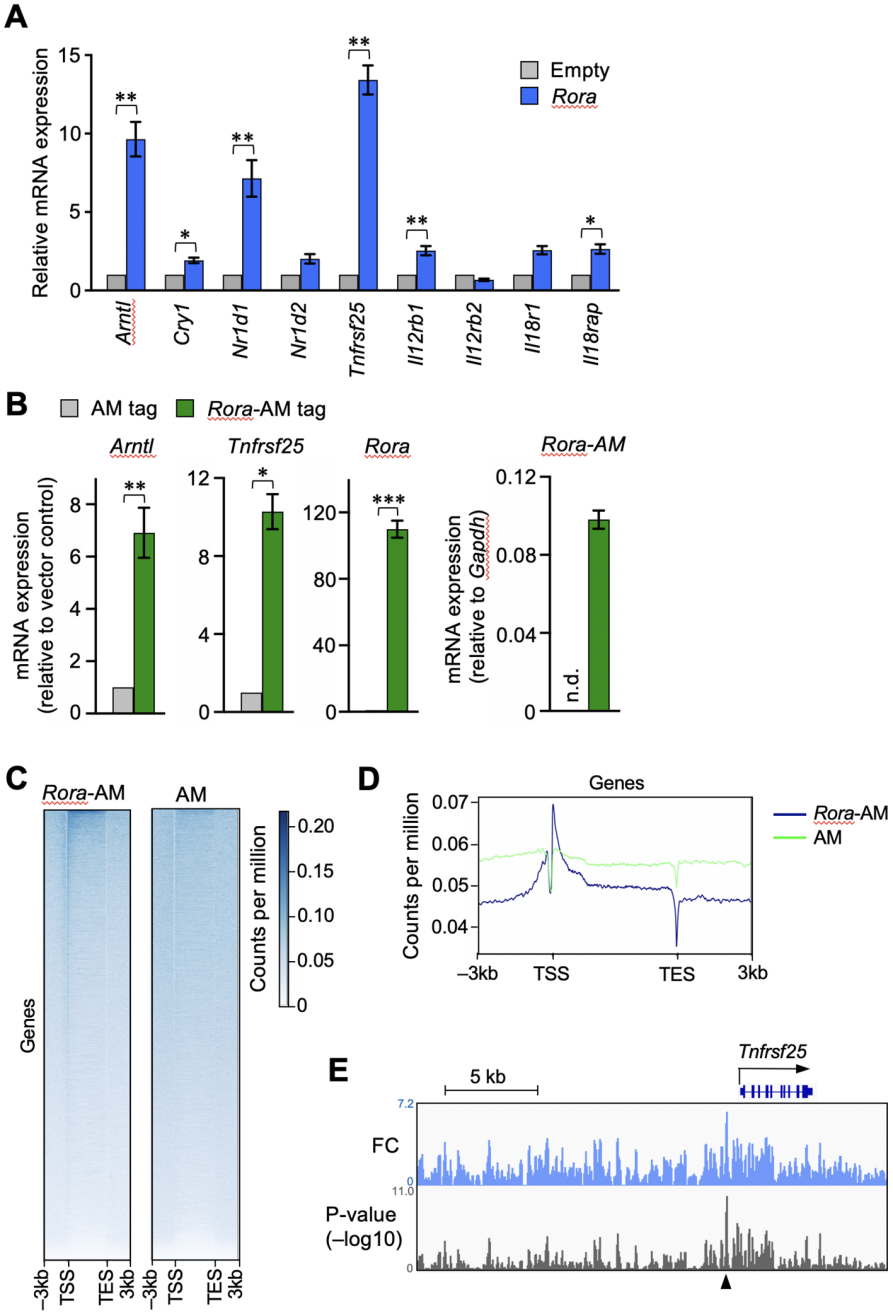
RORα transactivates the gene encoding DR3 in CD8^+^ T cells. RORα **(A)** or AM-tag–fused RORα **(B)** were retrovirally overexpressed in the OT-I T cells activated *in vitro*. The cells successfully infected with the retroviral vector were sorted based on the expression of green fluorescent protein. The mRNA expression of the indicated genes was analyzed by qPCR. The expression levels in the overexpressing cells are shown relative to those in the control cells transduced with empty **(A)** or AM-tag–expressing **(B)** vectors. Exceptionally, the green bar in the right end graph in *B* shows the expression of AM-tagged *Rora* relative to glyceraldehyde-3-phosphate dehydrogenase (*gapdh*) within the same samples, while no signals was obtained from the AM-tag-only control. *n.d.* denotes not detected. **(C, D)** The OT-I T cells overexpressing only the AM-tag or AM-tag–fused RORα were subjected to chromatin immunoprecipitation sequencing (ChIP-seq). The binding signals obtained from regions –3 to +3 kb surrounding the transcription start site (TSS) and transcription exit site (TES) at all genes. **(E)** IGV browser view near the *Tnfrsf25.* Peaks show fold changes (FC) and *p*-values over the tag-only control. ChIP-seq analysis was performed on sorted cell pellets pooled from three independent experiments **(C–E)**. Cumulative results from three **(B)** and four **(A)** independent experiments. Error bars, mean ± SEM. *P < 0.05, **P < 0.01 and ***P < 0.001 (paired Student’s *t*-test).

Next, we examined whether the enforced expression of RORα activates the transcription of *Tnfrsf25*. OT-I T cells were activated by antigen stimulation *in vitro* and then transduced with a retroviral vector expressing RORα. The expression of clock genes such as *Arntl*, *Nr1d1*, and *Cry1* was increased in the RORα-overexpressing T cells (**Figure 4A**), as expected from the results of shown in **Figure 3A**. Moreover, *Tnfrsf25* expression was significantly upregulated in these cells, far beyond increase observed in the clock genes (**Figure 4A**). Although the expression of genes encoding IL-12- and IL-18-receptor components was also upregulated, the extent was much lower than that observed in *Tnfrsf25*, *Arntl* and *Nr1d1* (**Figure 4A**).

The binding of RORα to the *Tnfrsf25* genomic region was assessed by ChIP-seq analysis. As commercially available anti-RORα antibodies did not work with ChIP-seq, RORα fused with Active Motif’s AM-tag was retrovirally overexpressed in activated CD8^+^ T cells. The mRNA expression of *Tnfrsf25* was upregulated in the cells expressing tagged RORα, but not in those containing the tag-only control vector (**Figure 4B**). The results of ChIP-seq using the anti-AM-tag antibody showed the enrichment of AM-tagged RORα, but not the AM-tag alone, near the transcription start sites of various genes (**Figures 4C, D**). To depict the RORα binding peaks, the fold changes and signal p-values were calculated over the vector control (**Figure 4E**). A remarkable peak was identified upstream of *Tnfrsf25*, as indicated by the arrowhead in **Figure 4E**. These results suggested that RORα activates *Tnfrsf25* transcription in postactivated CD8^+^ T cells.

### 3.4 Bystander activation is impaired in RORα-deficient memory CD8^+^ T cells *in vitro*

**Figure 5:**
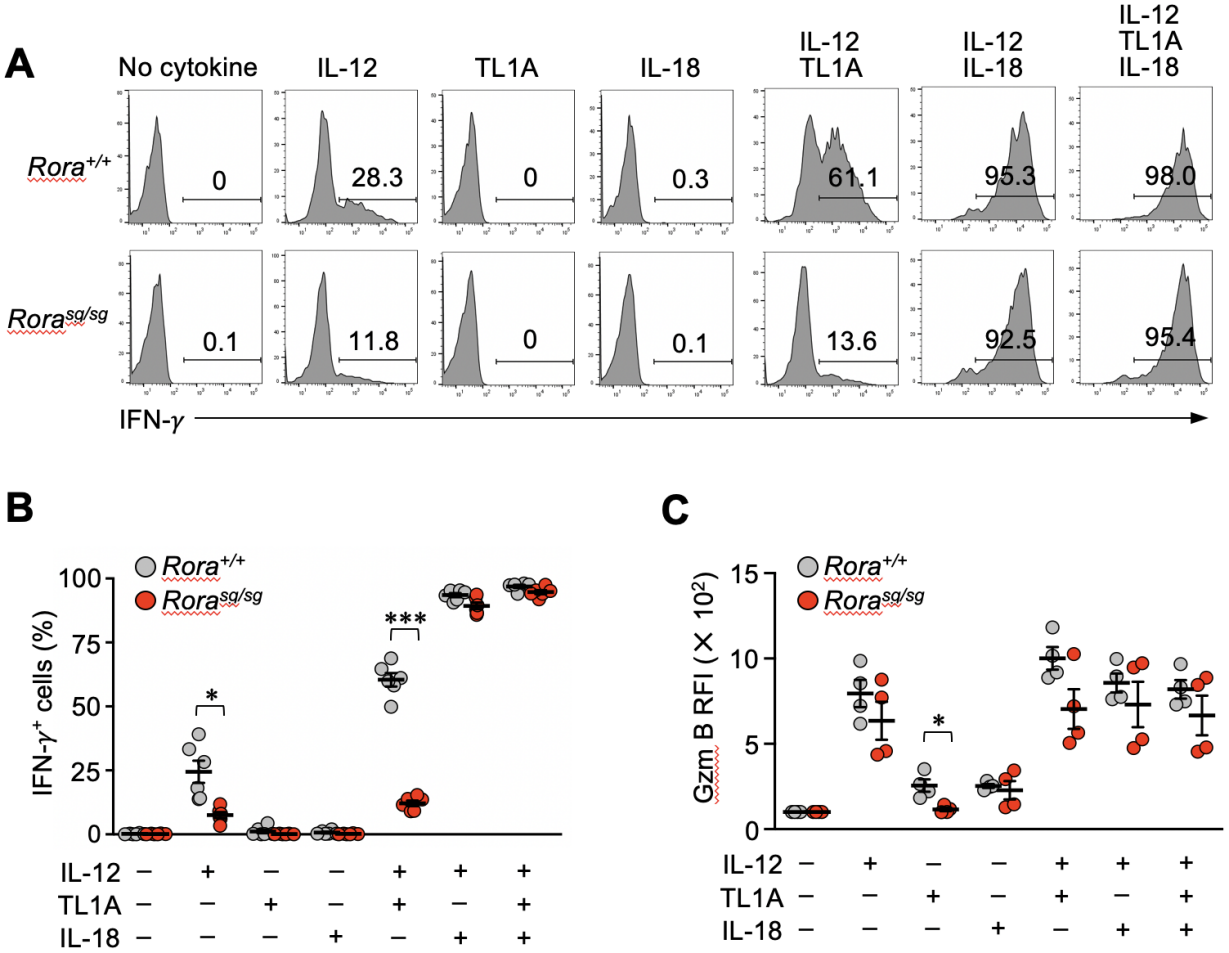
Bystander activation is impaired in the RORα-deficient memory CD8^+^ T cells *in vitro*. **(A–C)** Bone marrow chimeras were generated by transferring the bone marrow cells from *Rora^sg/sg^* and *Rora^+/+^* OT-I–transgenic littermates into the CD45-congenic recipients. Original donor-derived *Rora^sg/sg^* and *Rora^+/+^* naïve OT-I T cells were sorted from the spleens of the chimeras, and mice baring secondary memory OT-I T cells were induced by sequential T cell transfers and infections as shown in **Figure 1A**. Whole spleen cells obtained from these mice were cultured with the indicated cytokines (n = 6/group). **(A, B)** IFN-*γ* production was assessed via intracellular staining. **(C)** Granzyme B expression was assessed by intracellular staining and compared between the groups based on relative fluorescence intensity (RFI) normalized to the mean fluorescence intensity of the cells cultured without any cytokines. Representative of three **(A)** independent experiments. Cumulative results from two **(C)** or three **(B)** independent experiments. Error bars, mean ± SEM. *P < 0.05 and ***P < 0.001 (unpaired Student’s *t*-test).

TL1A can induce the bystander activation of effector CD4^+^ T cells synergistically with IL-12 and IL-18 (34, 35). Therefore, we next examined the IFN-γ production by *Rora^sg/sg^* secondary memory T cells *in vitro* after stimulating them with TL1A, IL-12, and IL-18 either individually or in combinations. Control *Rora^+/+^* secondary memory T cells stimulated with IL-12, but not TL1A and IL-18 alone, exhibited IFN-γ production (**Figures 5A, B**). Interestingly, *Rora^sg/sg^* T cells stimulated with IL-12 alone showed a moderate but substantial reduction in IFN-γ production in comparison with the *Rora^+/+^* T cells (**Figures 5A, B**). TL1A dramatically enhanced the bystander response to IL-12 in the *Rora^+/+^* T cells, although the synergistic effect of IL-12 and TL1A was milder than that of IL-12 and IL-18 (**Figures 5A, B**). Notably, the synergistic effect of TL1A and IL-12 was abrogated in the *Rora^sg/sg^* secondary memory T cells (**Figures 5A, B**), align with the earlier finding that RORα deficiency abolished DR3 expression in those cells (**Figures 3A–C**). The addition of IL-18 together with TL1A and IL-12 compensated for the lack of responsiveness to TL1A for bystander IFN-γ production (**Figures 5A, B**). As has been presented in **Supplementary Figures 2C, D**, IFN-γ production in response to the cognate antigen was comparable between the *Rora^+/+^* and *Rora^sg/sg^* secondary memory T cells and their descendant effector T cells, indicating that RORα deficiency did not affect the ability to produce IFN-γ. Bystander activation has been shown to induce granzyme B production from memory CD8^+^ T cells, promoting their cytotoxic functions (36). Stimulation with either IL-12 or IL-18 alone induced granzyme B equivalently in *Rora^+/+^* and *Rora^sg/sg^* secondary memory T cells, while stimulation with TL1A induced granzyme B only in the *Rora^+/+^* control cells (**Figure 5C**). This indicates RORα deficiency impairs TL1A-induced granzyme B production. Under the co-stimulation with IL-12 and TL1A, the granzyme B production tended to be slightly lower in the *Rora^sg/sg^* cells than in the *Rora^+/+^* cells, but this difference was not statistically significant (**Figure 5C**).

### 3.5 RORα-deficiency affects the bystander activation of memory CD8^+^ T cells *in vivo*

**Figure 6:**
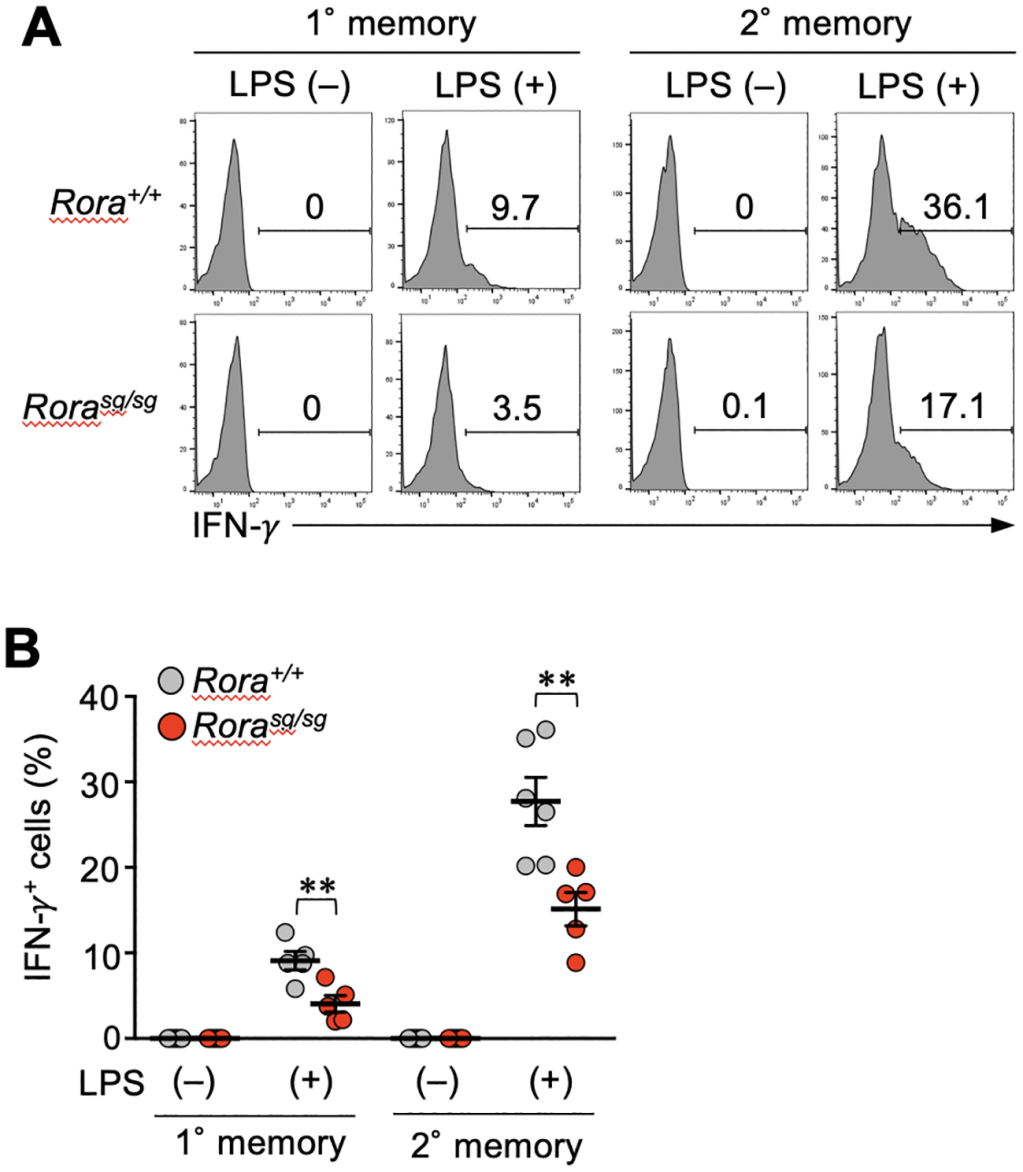
RORα-deficiency affects the bystander activation of memory CD8^+^ T cells *in vivo*. **(A, B)** Mice bearing *Rora^sg/sg^* and *Rora^+/+^* primary and secondary memory T cells were generated as described in the legend of **Figure 5**. These mice were intravenously injected with lipopolysaccharide (LPS). The spleens were harvested four hours later, and the IFN-γ production from the memory OT-I T cells was examined (n = 5–6/condition in each group) by intracellular staining and flow cytometry analysis. Representative **(A)** and cumulative **(B)** results of two independent experiments. Error bars, mean ± SEM. **P < 0.01 (unpaired Student’s *t*-test).

Finally, we examined whether RORα deficiency influences the bystander activation of memory CD8^+^ T cells *in vivo*. Systemic inflammation induced by the LPS of Gram-negative bacteria can stimulate bystander IFN-γ production in effector CD4^+^ T cells. This response is partially dependent on DR3 expression (37). Hence, the mice bearing *Rora^+/+^* or *Rora^sg/sg^* memory CD8^+^ T cells were intravenously injected with LPS, and four hours later, the IFN-γ production was detected in both primary and secondary memory T cells. In both *Rora^+/+^* and *Rora^sg/sg^* groups, the secondary memory T cells showed a greater response than the primary memory T cells (**Figures 6A, B**). The IFN-γ production was significantly diminished by RORα deficiency (**Figures 6A, B**). These results suggest that RORα regulates the bystander activation of memory CD8^+^ T cells *in vivo*.

## 4 Discussion

This study demonstrated the involvement of RORα in regulating bystander activation of memory CD8^+^ T cells. RORα acts as a transcription factor in inducing DR3 expression, primarily determining the reactivity of memory CD8^+^ T cells to TL1A (**Figures 3**, **4**). The synergistic effect of TL1A and IL-12 in inducing bystander activation in memory T cells was abrogated by RORα deficiency (**Figures 5A, B**). RORα has been shown to participate in molecular clock machinery. The transcriptional regulation of *Arntl*, the gene encoding Bmal1, by RORα is the essential for the circadian rhythm (21). However, RORα deficiency and overexpression in CD8^+^ T cells influenced the expression of *Tnfrsf25* more profoundly than *Arntl* (**Figures 3A**, **4A, B**). Our results suggest a tissue-specific function of RORα regulating the reactivity of T cells to inflammatory cues. While RORα regulates the reactivity of memory T cells to TL1A by inducing the TL1A receptor expression, the reason behind the partial but apparent impairment of the bystander response to IL-12 stimulation alone in RORα-deficient memory CD8^+^ T cells is unclear (**Figures 5A, B**). RORα deficiency did not affect the expression of genes encoding IL-12 receptor components (**Figure 3A**). It is possible that RORα-deficiency affects the memory CD8^+^ T cell response to unknown factors produced by CD4^+^ T cells, because IL-12 also activates effector and memory CD4^+^ T cells in bystander manner (11, 35). Alternatively, RORα may involved in the pathways downstream of the IL-12 receptor, including the expression of molecules associated with the intracellular signaling.

The synergistic effect of IL-12 and IL-18 in inducing bystander activation is well recognized, and these cytokines are widely used to evaluate the bystander response *in vitro*. TL1A is also a proinflammatory cytokine, like IL-12 and IL-18, but its role in bystander activation has been poorly characterized in CD8^+^ T cells, besides a study that comprehensively tested various cytokines that induced bystander activation (38). RORα deficiency abrogated the bystander response of secondary memory T cells stimulated using a combination of IL-12 and TL1A *in vitro* (**Figures 5A, B**). On the other hand, the response to LPS-driven inflammation was also remarkably impaired in RORα-deficient memory T cells, demonstrating the involvement of RORα in bystander activation *in vivo* (**Figures 6A, B**). However, this result does not necessarily indicate that RORα regulates the TL1A-driven bystander activation *in vivo*, because LPS induces a variety of inflammatory cytokines (37) and the possibility remains that RORα affects the response to any of them. The involvement of TL1A in bystander activation of CD8^+^ T cells *in vivo* should be directly addressed in the future.

We observed that the DR3 expression in pathogen-specific CD8^+^ T cells increased over time after primary infection, peaking in the memory phase (**Figures 3B, C**). Secondary memory CD8^+^ T cells also exhibited high DR3 levels, which were heavily dependent on elevated RORα in these cells (**Figures 1F**, **3B, C**). Compared with that observed in secondary memory T cells, DR3 expression in primary memory T cells was less affected by RORα deficiency (**Figures 3B, C**), suggesting the involvement of other factors regulating DR3 expression in primary memory T cells.

Repeated antigen encounters shift the ability of memory CD8^+^ T cells toward enhancing the immediate immune response (17, 31, 39), primarily by altering the memory CD8^+^ T cell subsets. The memory CD8^+^ T cell pool is composed of heterogeneous subpopulations (40, 41). A previous study showed that the bystander activation occurring in the CD62L^lo^ cells were more profound than that in CD62L^hi^ memory CD8^+^ T cells (10, 18). Repeated antigen encounters increase the proportion of memory CD8^+^ T cells with effector-like characteristics, including CD62L^lo^ and KLRG1^hi^ phenotypes, high killing activity, and terminal differentiation (31, 39, 42). An increase in bystander activation capacity has been shown to correlate with the heightened expression of IL-12 and IL-18 receptors (17). Similarly, in our experiments, secondary memory CD8^+^ T cells enriched with the KLRG1^+^ effector-like subpopulation showed a greater bystander response than primary memory T cells (**Figures 1E**, **6A, B**), which was accompanied by elevated RORα expression and RORα-dependent DR3 expression (**Figures 1F**, **3B, C**). This might be the underlying reason behind the increase in the immediate protective capacity according to the antigen experience history. Meanwhile, TL1A has been linked with the pathogenesis of inflammatory and autoimmune diseases (35, 43–45). Elucidating how memory T cells are functionally altered through repetitive immunization would help design effective and safe vaccination regimens.

Ge et al. showed that bystander-activation of tissue-resident memory CD8^+^ T cells boosts neutrophil recruitment to the infection site, counteracting of noncognate bacterial infections (9). Furthermore, circulating memory CD8^+^ T cells with irrelevant antigen specificity have been shown to be recruited to the infected tissue, followed by bystander activation in the inflammatory environment (13, 18). Most secondary memory CD8^+^ T cells are composed of a memory subset called long-lived effector cells (31, 46) that largely overlap with the terminally differentiated memory CD8 T cell population recently characterized in detail by Milner et al. (47). This memory T cell subset preferentially localizes in the peripheral blood, tertiary tissues, and splenic red pulp and expresses high levels of CX3C chemokine receptor 1 associated with immune cell trafficking to the infection site (31, 46, 47). Therefore, secondary memory CD8^+^ T cells enriched with long-lived effector cells may exert the immediate protective function by quickly migrating to the infection site. The heightened sensitivity of secondary memory T cells to the inflammatory cues, such as IL-12, IL-18, and TL1A, resulting in IFN-γ production via bystander activation, can potentially condition the infection site for efficient immune responses before antigen recognition and antigen-specific killing.

Contrary to its role in facilitating host defense, bystander activation can also mediate the unwanted immune responses. Bystander activation of effector CD4^+^ T cells exacerbates inflammatory bowel disease and rheumatoid arthritis via TL1A-mediated production of IFN-γ and IL-17 synergistically with pro-inflammatory cytokines such as IL-12, IL-18 and IL-23 (35, 44). Currently, RORα has been shown to be, at least partly, involved in inflammatory diseases by transactivating IL-17. Furthermore, an inverse agonist of RORα was shown to effectively alleviate autoimmune encephalomyelitis and colitis in mice (48). If our finding that RORα regulates the TL1A responsiveness and bystander activation in CD8^+^ T cells can be extended to CD4^+^ T cells, it may reveal a novel disease pathogenesis through RORα and pave the way for a therapeutic strategy to reduce disease-associated bystander activation by inhibiting RORα. Further investigations in CD4^+^ T cells are required.

In conclusion, we found that RORα regulates the bystander activation of memory CD8^+^ T cells, at least partially, through the transactivation of DR3, an inflammatory cytokine receptor. RORα expression was markedly elevated in secondary memory T cells, and their DR3 expression was heavily dependent on RORα. These findings provide molecular insights into how the immune system develops immediate protective capacities through repeated infections or vaccinations.

## Data Availability

The data presented in the study are deposited in the NCBI GEO repository, accession number GSE304146 and GSE304290.
