# Supplementary material for: Retinoic acid receptor-related orphan receptor α regulates bystander activation of memory CD8+ T cells
Source: Front Immunol. 2025 Aug 14;16:1647746. doi: 10.3389/fimmu.2025.1647746 (PMC12392277; doi:10.3389/fimmu.2025.1647746)
Supplement: Supplementary file 1 [file DataSheet1.docx]

Supplementary Material

# Supplementary Figures and Tables

## Supplementary Figures

##
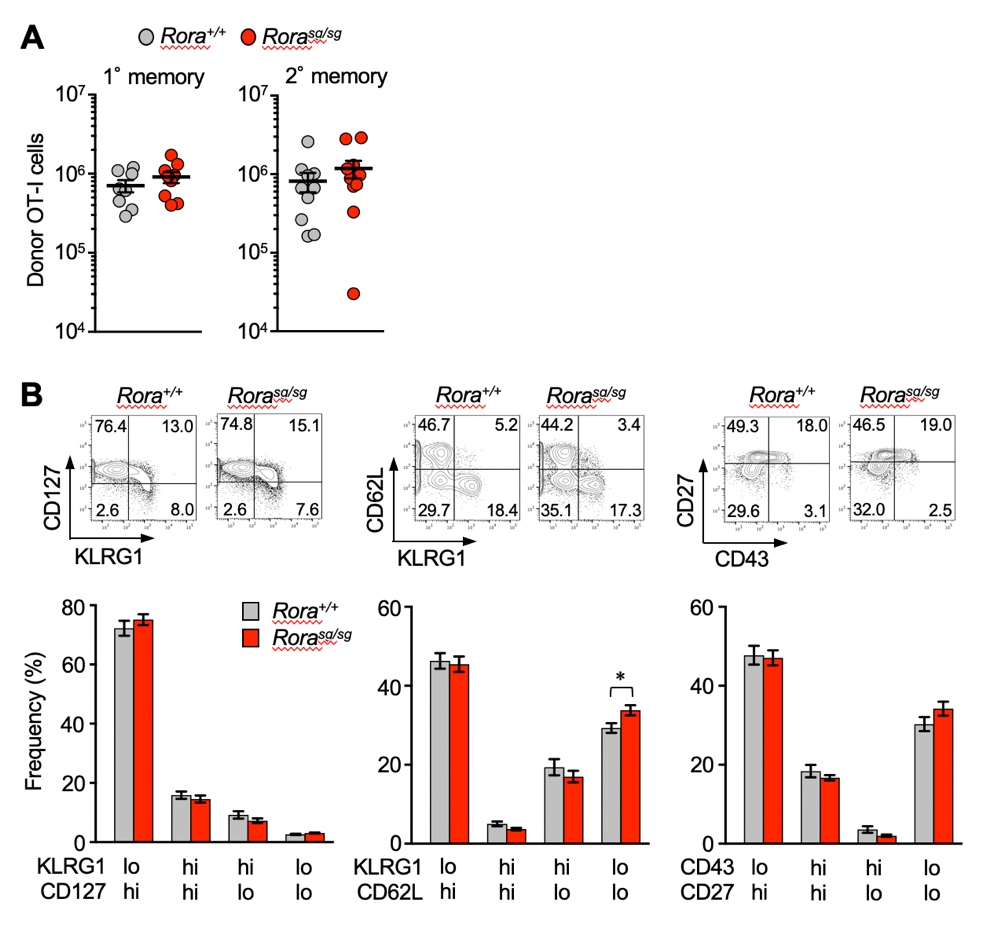


## Supplementary Figure 1. Overview of the RORα-deficient memory CD8^+^ T cells. Bone marrow chimeras were generated by transferring the bone marrow cells from *Rora^sg/sg^* and *Rora^+/+^* OT-I–transgenic littermates into the CD45-congenic wild-type recipient mice. Then, *Rora^sg/sg^* and *Rora^+/+^* naïve OT-I T cells were isolated from the spleens of these bone marrow chimeras. Primary and secondary memory T cells were induced as shown in Fig. 1A. (A) The number of primary (n = 8–9/group) and secondary (n = 10/group) memory T cells in the spleen of recipient mice. (B, C) Phenotypic analysis of primary memory T cells based on the indicated surface markers (*Rora^+/+^*, n = 8; *Rora^sg/sg^*, n = 9). Cumulative results from two (B) and three (A) independent experiments. Error bars, mean ± SEM. **P* < 0.05 (unpaired Student’s *t*-test).


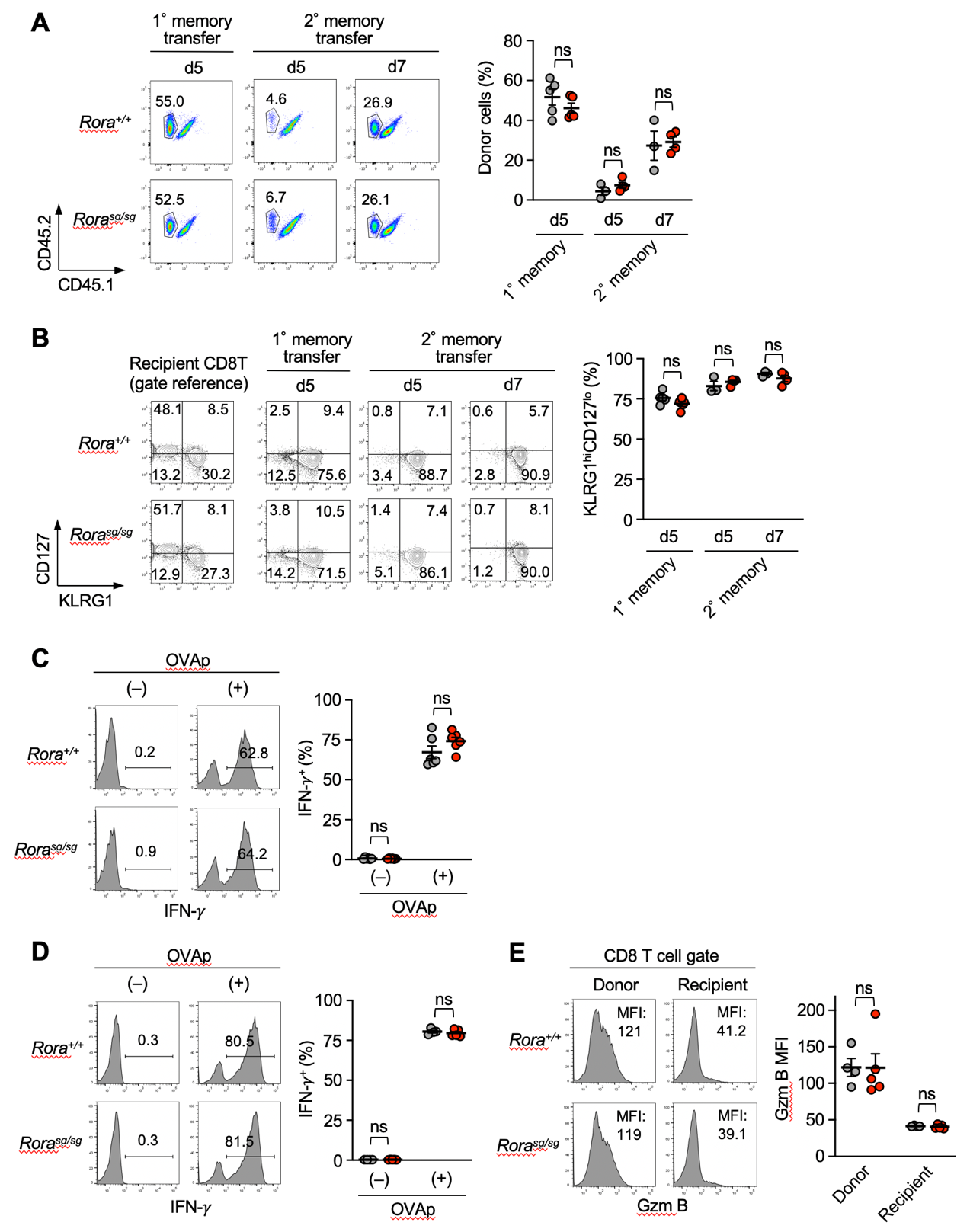


## Supplementary Figure 2. Antigen-specific recall responses of the RORα-deficient memory CD8^+^ T cells. Bone marrow chimeras were generated by transferring the bone marrow cells from *Rora^sg/sg^* and *Rora^+/+^* OT-I–transgenic littermates (CD45.1^–^CD45.2^+^) into CD45-congenic recipients (CD45.1^+^CD45.2^+^). Original donor-derived *Rora^sg/sg^* and *Rora^+/+^* naïve OT-I T cells were sorted from the spleens of the chimeras based on the expression of CD45.1 and CD45.2. Primary or secondary memory OT-I T cells were induced by sequential transfers and LM-OVA infections as shown in Fig. 1A. (A, B) The primary or secondary memory OT-I T cells were obtained by sorting the original donor-derived CD8^+^ T cells from the spleens based on the CD45 markers. The memory OT-I T cells were further transferred into new recipients (CD45.1^+^CD45.2^+^) followed by infection with LM-OVA. The blood was obtained from the recipients transferred with the primary memory OT-I cells at day 5 postinfection. Donor OT-I T cells were analyzed for the frequency within CD8^+^ gate (A) and phenotype (B). Similarly, the recipients transferred with the secondary memory OT-I cells were analyzed at day 5 and 7 post infection (n = 3–5/group). Expression of CD127 and KLRG1 in recipient CD8^+^ T cells is also shown as a gate reference in *B*. (C) *In vitro* effector response of secondary memory T cells. Whole splenocytes from the mice bearing the secondary memory OT-I T cells were stimulated with OVA peptide (OVAp). IFN-γ production by the donor OT-I T cells was assessed by intracellular staining (n = 6/group). (D, E) *In vivo* effector response of secondary memory T cells. Isolated secondary memory OT-I T cells were further transferred to new recipients followed by infection with LM-OVA (n = 4–5/group). Seven days later, whole spleen cells obtained from the recipients were stimulated with OVAp and analyzed for IFN-γ production by the donor OT-I T cells (D). Granzyme B expression in the donor OT-I T cells was assessed similarly without further *in vitro* stimulation (E). Cumulative results from two (A–E) independent experiments. Error bars, mean ± SEM. “ns” denotes “not significant”.


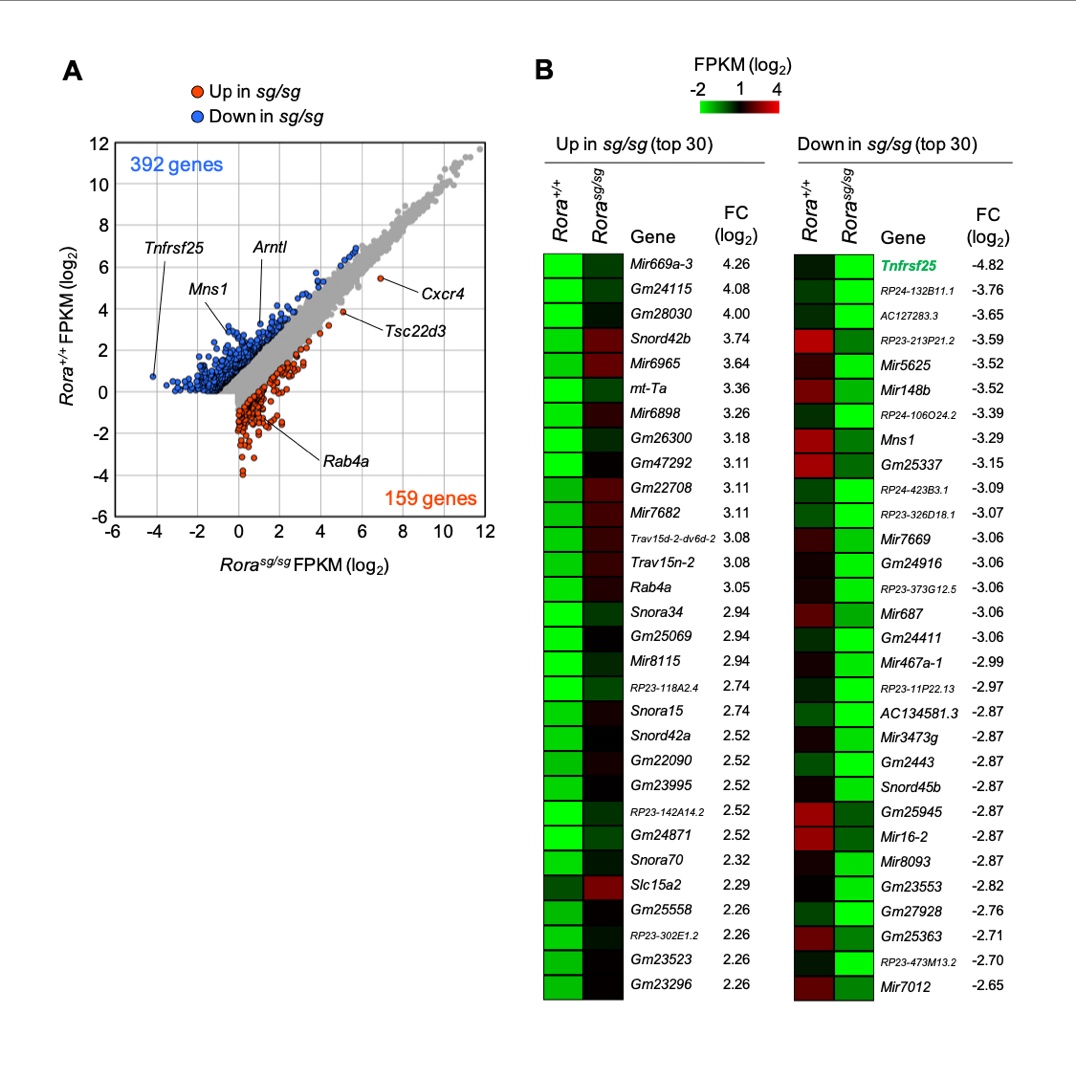


## Supplementary Figure 3. RNA-sequencing analysis at day 7 post-infection. Naïve *Rora^+/+^* and *Rora^sg/sg^* OT-I T cells obtained from the bone marrow chimeras were transferred into CD45-congenic recipient mice (2 × 10^4^ cells per mouse). On the following day, the recipient mice were infected with an *ActA*-deficient attenuated strain of *Listeria monocytogenes* expressing ovalbumin (5 × 10^6^ colony-forming units per mouse). KLRG1^+^ donor OT-I T cells were sorted from the splenocytes of recipients at day 7 post-infection (n = 3/group). The RNA isolated from the pooled cells was subjected to RNA-sequencing analysis. (A) The scatter plot was drawn based on the FPKM values from one mouse in each group. The cutoff FPKM value was set to 1. The genes exhibiting ≥ twofold expression in *Rora^+/+^* cells (blue dots) or *Rora^sg/sg^* cells (red dots) are highlighted. (B) The heatmaps of the upregulated and downregulated genes in the *Rora^sg/sg^* T cells. The data of the top 30 genes based on the fold change (FC) is shown.


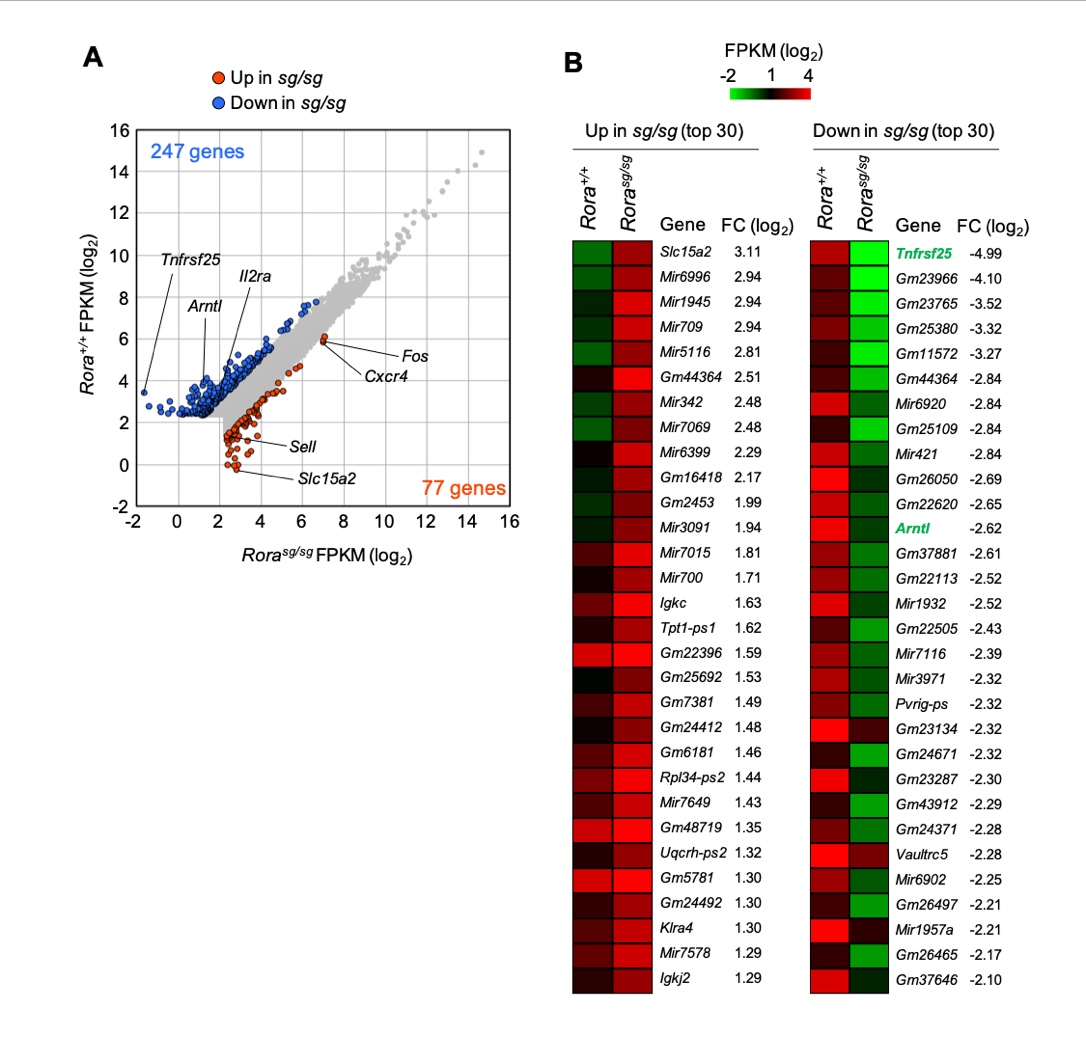


**Supplementary Figure 4.** RNA-sequencing analysis at day 10 post-infection. Naïve *Rora^+/+^* and *Rora^sg/sg^* OT-I T cells obtained from the bone marrow chimeras were transferred into CD45-congenic recipient mice (2 × 10^4^ cells per mouse). On the following day, the recipient mice were infected with an *ActA*-deficient attenuated strain of *Listeria monocytogenes* expressing ovalbumin (5 × 10^6^ colony-forming units per mouse). KLRG1^+^ donor OT-I T cells were sorted from the splenocytes of recipients at day 10 post-infection (n = 3/group). The RNA isolated from the pooled cells was subjected to RNA-sequencing analysis. **(A)** The scatter plot was drawn based on the FPKM values from one mouse in each group. The cutoff FPKM value was set to 5. The genes exhibiting ≥ twofold expression in *Rora^+/+^* cells (blue dots) or *Rora^sg/sg^* cells (red dots) are highlighted. **(B)** The heatmaps of the upregulated and downregulated genes in the *Rora^sg/sg^* T cells. The data of the top 30 genes based on the fold change (FC) is shown.

## Supplementary Tables

## Supplementary Table 1. Primers used in quantitative PCR.

| Primer | Sequence |
| --- | --- |
| *Gapdh* F | 5’-TTGTCAGCAATGCATCCTGCAC-3’ |
| *Gapdh* R | 5’-GAAGGCCATGCCAGTGAGCTTC-3’ |
| *Rora* F | 5’-ATTCAGCTGGCCCTTCAGCAC-3’ |
| *Rora* R | 5’-TGGAGGAAAATGGAGTCGCACAATG-3’ |
| *Tnfrsf25* F | 5’-CATGTCTGGCAGGTGTGACT-3’ |
| *Tnfrsf25* R | 5’-ACTTTGCCGAGCAGTTCTCA-3’ |
| *Arntl* F | 5’-CCTAATTCTCAGGGCAGCAGAT-3’ |
| *Arntl* R | 5’-TCCAGTCTTGGCATCAATGAGT-3’ |
| *Cry1* F | 5’-GCATGACCCCTCTGTCTGAT-3’ |
| *Cry1* R | 5’-AGGGAGTTTGCATTCATTCG-3’ |
| *Nr1d1* F | 5’-CATGGTGCTACTGTGTAAGGTGTGT -3’ |
| *Nr1d1* R | 5’-GATTGATGCGAACGATGGAGCAGTT -3’ |
| *Nr1d2* F | 5’-CCCAAGAACGCTGATATCTCTAG-3’ |
| *Nr1d2* R | 5’-ACACAGTAGAACCATGCCAC-3’ |
| *Il12rb1* F | 5’-ATGCAAGGACAGTCACCACA -3’ |
| *Il12rb1* R | 5’-CGGAGAGGTTCAGCTTCTTG-3’ |
| *Il12rb2* F | 5’-AGTGGCCCTCACATTACTGC-3’ |
| *Il12rb2* R | 5’-GGCTGTAGGCTGCTTATTGG-3’ |
| *Il18r1* F | 5’-GCAAACTGCATGCTTCAAAA-3’ |
| *Il18r1* R | 5’-AGGCGAGAACAAGCACAGTT-3’ |
| *Il18rap* F | 5’-GAGGAACACCTGGCTCTGAA-3’ |
| *Il18rap* R | 5’-AGGCAAGATTCACTGCTGCT-3’ |
| *Rora-AM* F | 5’-CAGACATTGTGCGACTCCAT-3’ |
| *Rora-AM* R | 5’-GCCGCCTAGTAAGCCTGAG-3’ |
